# Quantifying Gene Essentiality Based on the Context of Cellular Components

**DOI:** 10.3389/fgene.2019.01342

**Published:** 2020-01-21

**Authors:** Kaiwen Jia, Yuan Zhou, Qinghua Cui

**Affiliations:** Department of Biomedical Informatics, Department of Physiology and Pathophysiology, Center for Noncoding RNA Medicine, MOE Key Lab of Cardiovascular Sciences, School of Basic Medical Sciences, Peking University, Beijing, China

**Keywords:** cellular components, localization diversity, gene characteristic, gene essentiality, drug target

## Abstract

Different genes have their protein products localized in various subcellular compartments. The diversity in protein localization may serve as a gene characteristic, revealing gene essentiality from a subcellular perspective. To measure this diversity, we introduced a Subcellular Diversity Index (SDI) based on the Gene Ontology-Cellular Component Ontology (GO-CCO) and a semantic similarity measure of GO terms. Analyses revealed that SDI of human genes was well correlated with some known measures of gene essentiality, including protein–protein interaction (PPI) network topology measurements, dN/dS ratio, homologous gene number, expression level and tissue specificity. In addition, SDI had a good performance in predicting human essential genes (AUC = 0.702) and drug target genes (AUC = 0.704), and drug targets with higher SDI scores tended to cause more side-effects. The results suggest that SDI could be used to identify novel drug targets and to guide the filtering of drug targets with fewer potential side effects. Finally, we developed a user-friendly online database for querying SDI score for genes across eight species, and the predicted probabilities of human drug target based on SDI. The online database of SDI is available at: http://www.cuilab.cn/sdi.

## Introduction

After gaining the potential genes of interest from a large-scale screen, it comes to the need to determine which particular gene is worthy of future research. By linking the genes of interest and their functional annotations together, the enriched functions of interest can be found and evaluated. On the other hand, when considering the gene per se, important characteristics can be used as references to assess the status of each gene of interest, in different dimensions, in the entire genome.

At a genomic level, the idea to minimize the genome into “the right size” led to the notion of gene essentiality and essential genes (Maniloff, 1996). Essential genes were defined as those genes which are indispensable for reproductive success, and that loss of their function compromises viability of the individual (Bartha et al., 2017; Rancati et al., 2017). Nowadays, multiple essential genes have been identified by gene deletion technologies in genome-scale across organisms and cell types (Wang et al., 2015; Evers et al., 2016; Morgens et al., 2016). On the other hand, computational methods have revealed that gene essentiality is correlated with other measurements of a gene, such as the sequence conservation, the protein–protein interaction (PPI) network topology and the expression patterns (Liang and Li, 2007; Deng et al., 2010; Zhao et al., 2016).

For a cell, proteins must be transported to the appropriate subcellular locations in order to provide the context support for their biological functions. The subcellular mislocalization of human proteins can cause severe diseases such as Alzheimer's disease, kidney stones and cancer (Hung and Link, 2011). The subcellular localization diversity of the protein products of a gene may serve as an important characteristic in revealing gene essentiality, in the context of subcellular locations. Previous studies have discussed the protein localization specificity in prokaryotes (Peng and Gao, 2014), and integrated the subcellular localized information with PPI topology for predicting essential genes (Acencio and Lemke, 2009; Li et al., 2016; Li et al., 2018), using annotation data from the Gene Ontology-Cellular Component Ontology (GO-CCO) (Ashburner et al., 2000). Although the priority of these components definitely needs to be discussed, another potentially important characteristic of protein localization seems to be ignored, which is the subcellular diversity. Proteins that occur in a wild range of components may be more essential than proteins with a unique component membership.

In this work, we presented an algorithm to calculate a Subcellular Diversity Index (SDI), and it was applied to genes in eight species based on the GO-CCO. SDI can be used to assess subcellular diversity of a gene, and qualitatively speaking, higher SDI of a gene means it has its products present in a greater diversity of cellular components. SDI can be calculated directly for all GO-CC-annotation-covered genes, and can be ranked and compared at a genomic level. Analysis result for SDI suggests that SDI can quantify gene essentiality, and can be used as a new characteristic for assessing gene potential in various aspects. SDI also showed its usefulness in predicting novel drug targets and those with fewer drug side effects.

## Materials and Methods

### Calculating the SDI for Genes in Eight Species

We used GO-CCO to reflect subcellular diversity of genes. The information of GO-CC terms is available for most genes across species and has a large deviation between different genes, making this data preferable for reflecting subcellular diversity. As GO-CCO is structured as a directed acyclic graph (DAG), there may be inheritance relationships between the annotated terms of each gene. The inheritance relationships can lead to certain overlap in the information of all annotated terms, and in this case, the overlap information reveals redundant quantity of localization information. The SDI is calculated based on the quantification and removal of the redundancy in GO-CC terms of each gene.

We presented the following equation to calculate the SDI of a gene:

$$SDI=\left(1-p_{1}\right)+\left(1-p_{2}\right)+\cdots +\left(1-p_{n}\right)$$

where

$$p_{i\left(i\in c\right)}=\{\begin{array}{r} 1,n=1\\ \sqrt{\frac{s_{1}^{2}+s_{2}^{2}+\cdots +s_{n-1^{2}}}{n-1}},n>1 \end{array}$$

For each gene, a cluster of all GO-CC terms in the annotation file was referred as *c*, and the number of terms in *c* was referred as *n* (terms in all evidence codes were included, repetitive terms was only counted once). Then, for each term *i* in *c*, a penalty score *p_i_* was calculated to estimate the similarity to all other terms. If *n* = 1, the penalty score equals to 1. If *n* > 1, a similarity score *s* (ranges from 0 to 1) was calculated for *i* and all other terms in *c* one by one, using the semantic similarity measure of GO terms from Wang et al. (2007), and calculated for a Root Mean Square (RMS) as *p_i_*.

We chose Wang's method because it is not only node-based or edge-based but a hybrid method, and it is widely used and proved to be strong in a decade. Wang's method was compared to a more recent and well-conducted method, TCSS (Jain and Bader, 2010), and the result showed a slight difference (**Figure S1**). The RMS was used because applying RMS in the SDI algorithm gave the best performance in validating human essential genes, compared with the arithmetic mean or the geometric mean (**Figure S2**).

We applied the above algorithm to calculate gene SDI for eight species by writing a Python script. Representing the structure of the GO-CCO, a GO-basic file was read in as a directed acyclic graph (DAG) using Python package NetworkX (Hagberg et al., 2008). When implementing Wang's method, we tested a few groups of different contribution factors for “is-a” and “part-of” relations in GO-CCO in human data (**Table S1**). As the results only showed slight differences, we chose 0.8 for “is-a” and 0.6 for “part-of” as the authors recommended in the article.

The GO-basic (OBO) file was obtained from the Gene Ontology Consortium (http://www.geneontology.org/page/download-ontology) (Ashburner et al., 2000). The GO annotation files for eight species were obtained from National Center for Biotechnology Information (NCBI, http://ftp.ncbi.nih.gov/gene/DATA/gene2go.gz) (NCBI, 2016).

### The Correlation Between SDI of Human Genes and Other Known Measures of Gene Essentiality

The dN/dS ratio (the ratio between nonsynonymous substitutions rate and synonymous substitutions rate) dataset for each human–mouse homolog was derived from the Ensembl database (release 83) (Aken et al., 2016) to estimate the balance between neutral mutations, negative selection mutations and positive selection mutations on homologous genes. The homologous gene number dataset was obtained from the Homologene database (build 68) (NCBI, 2016). The expression level and tissue specificity for each gene were calculated based on the data from Su et al. (2004). The human and mouse PPI network was downloaded from the BioGRID database (build 3.4.140), with the deletion of links including non-human or non-mouse proteins, respectively (Chatr-aryamontri et al., 2017), and the network degree and betweenness for each gene node were calculated using Python package NetworkX (Hagberg et al., 2008). Spearman's correlation tests were carried out between SDI and dN/dS ratio, homologous gene number, expression level, tissue specificity, PPI degree and PPI betweenness.

### Sensitivity Test and Comparison Test for the Performance of SDI in Predicting Essential Genes and Drug Targets

The human essential genes (2,501) and essential genes of the other six species were derived from the DEG database (version 10.6) (Luo et al., 2014). For unbiased essential gene datasets, 4,426 essential genes from Hart et al.’s CRISPR screens data (Hart et al., 2015) were derived from DEG database (version 15.2), and 2,132 essential genes from Cancer Dependency Map were downloaded from the DepMap website (https://depmap.org/portal/depmap/) (Tsherniak et al., 2017). The drug target genes (approved, 2,682) were downloaded from Drugbank (release 5.0.10) (Wishart et al., 2018).

The receiver operating characteristic (ROC) curves for validating the essential genes and drug targets and calculation for the Area Under Curve (AUC) were performed by R package pROC (Robin et al., 2011). The binary logistic regression models and 10-fold cross-validations were performed using R package caret (Kuhn, 2008). For comparison tests, the Pearson Chi-squared tests were performed to compare the fraction of essential genes and drug targets in 10 equally divided groups ranked by SDI.

### The Relationship Between SDI and Side-Effects in Drug Targets

The active drug targets (approved, pharmacologically active, 821) and the ID—drug name transformation file were obtained from DrugBank (Wishart et al., 2018). The information of side-effects of the corresponding drugs were obtained from the SIDER database (Kuhn et al., 2015) and VigiAccess (Shankar, 2016). Terms including “product issues” and its sub terms were filtered for data in VigiAccess. As a result, 369 and 712 drug targets were mapped to drug side-effect terms from SIDER and VigiAccess, respectively. Spearman's correlation tests were carried out between SDI and number of terms of side-effects of the corresponding drug targets.

### Data Availability

A web server was built for querying SDI of genes in eight species, and the predicted probabilities of human drug target from a binary logistic regression model based on SDI (www.cuilab.cn/sdi). The source code (running in Python 2.7) for calculating SDI and all data in the database are available in the web server.

## Results

### The SDI

**Figure 1:**
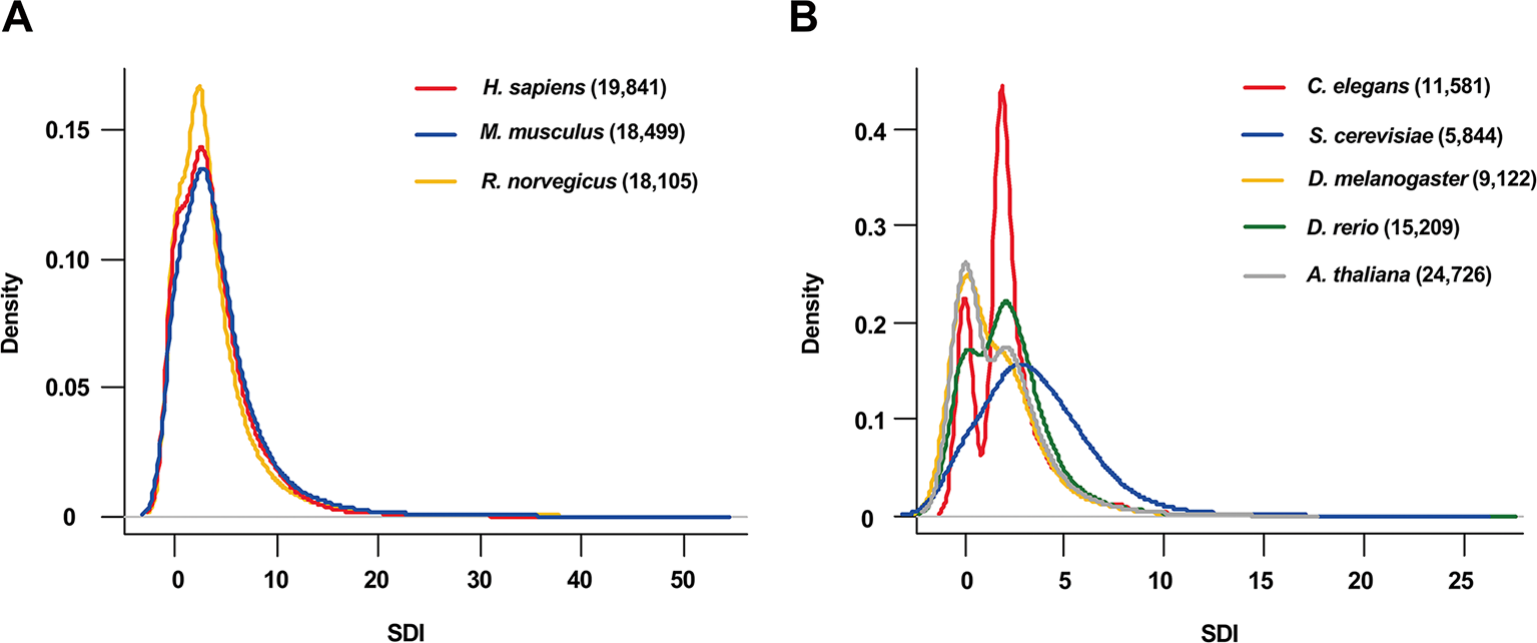
The distribution of SDI in eight species. The density plots show the distribution of SDI in thee mammals (Bandwidth = 0.9134) **(A)** and 5 other species (Bandwidth = 0.4149) **(B)**. Gene number for each species is presented in the brackets. *C.elegans* has a larger proportion of genes with two GO-CC terms than other species which may lead to its bimodal distribution (**Table S2**). SDI, Subcellular Diversity Index; GO-CC, Gene Ontology-Cellular Component.

The SDI was calculated for 122,435 genes in eight species, including *Homo sapiens, Mus musculus, Rattus norvegicus, Drosophila melanogaster, Caenorhabditis elegans, Danio rerio, Arabidopsis thaliana and Saccharomyces cerevisiae* (**Figure 1**, **Table 1**). As the distribution of SDI was slightly different across species, we examined the total GO-CC term counts for each gene in all eight species, and calculated for a percentage of the number of genes with one to five GO-CC terms to the total number of genes in each species (**Table S2**). Homolog genes in human and mouse have highly similar rank of SDI scores (14,647 genes, Spearman's correlation test: ρ = 0.83, P = 0). The top 30 genes with high SDI scores have functions mainly related to endocytosis and transendothelial migrations. Among these genes, two genes (*DNM2*, *NUMA1*) are listed in three essential gene datasets, five genes (*CTNNB1*, *ITGB1*, *EGFR*, *CALR*, *CDH2*) are listed in two essential gene datasets, and 11 genes are listed in one essential gene dataset (**Table 2**). For those genes not listed in three essential gene datasets, they also play important roles in severe diseases. The first-ranked gene *FMR1* was proved to be associated with mental diseases like mental retardation and autism (Mila et al., 2018), and five other genes (*APP, SNCA, MAPT, SIRT2, APOE*) are known to be associated to Alzheimer's disease. We mainly used human genes to further analyze the correlation of SDI and gene essentiality at a genomic level.

**Table 1:** Statistical summary for SDI.

Species	Minimum	The 1^st^ Quartile	Median	Mean	The 3^rd^ Quartile	Maximum	Gene Number
**Mammals**							
*H. sapiens*	0	1.455	2.875	3.577	4.911	44.386	19,341
*M. musculus*	0	1.707	2.950	3.966	5.532	51.211	18,499
*R. norvegicus*	0	1.401	2.502	3.393	4.622	51.216	18,105
**Others**							
*C. elegans*	0	1.471	1.848	2.116	2.809	25.609	11,581
*S. cerevisiae*	0	1.861	2.959	3.436	4.847	23.078	5,844
*D. melanogaster*	0	0	1.318	1.549	2.540	22.688	9,122
*D. rerio*	0	0	1.848	2.083	2.924	25.247	15,209
*A. thaliana*	0	0	1.588	1.680	2.814	15.486	24,726

The SDI scores show three digits after the decimal point. SDI, Subcellular Diversity Index.

**Table 2:** Top 30 human genes with the highest SDI scores.

Gene Symbol	SDI	SDI Ranking	DEG	Hart et al.	DepMap	Drug Target
FMR1	44.386	1				
EZR	40.349	2	A			
LRRK2	40.098	3	A			
CTNNB1	37.571	4	A	A		A
APP	36.614	5				A
DNM2	35.516	6	A	A	A	
HSPA8	34.595	7		A		A
ANXA2	31.624	8				A
ITGB1	31.343	9	A	A		A
NUMA1	29.691	10	A	A	A	
DLG1	29.585	11	A			
SNCA	29.443	12				A
ANXA1	28.637	13				A
DAG1	27.765	14	A			
MAPT	27.476	15				A
VAMP2	27.233	16	A			A
JUP	26.783	17	A			A
EGFR	26.671	18	A	A		A
RHOA	26.617	19		A		
STX4	26.568	20	A			
GPER1	26.46	21				A
SIRT2	26.33	22				
CALR	26.25	23	A	A		A
CDH2	25.761	24	A	A		
DLG4	25.716	25	A			A
PSEN2	25.685	26				
ANK3	25.627	27				
HSP90AB1	25.607	28	A			
APOE	25.59	29				A
RAB5A	25.373	30				

The SDI scores show three digits after the decimal point. “A” means this gene is available in this particular essential gene or drug target gene list. SDI, Subcellular Diversity Index.

### The Correlation Between SDI and Other Essentiality Measures of Human Genes

**Figure 2:**
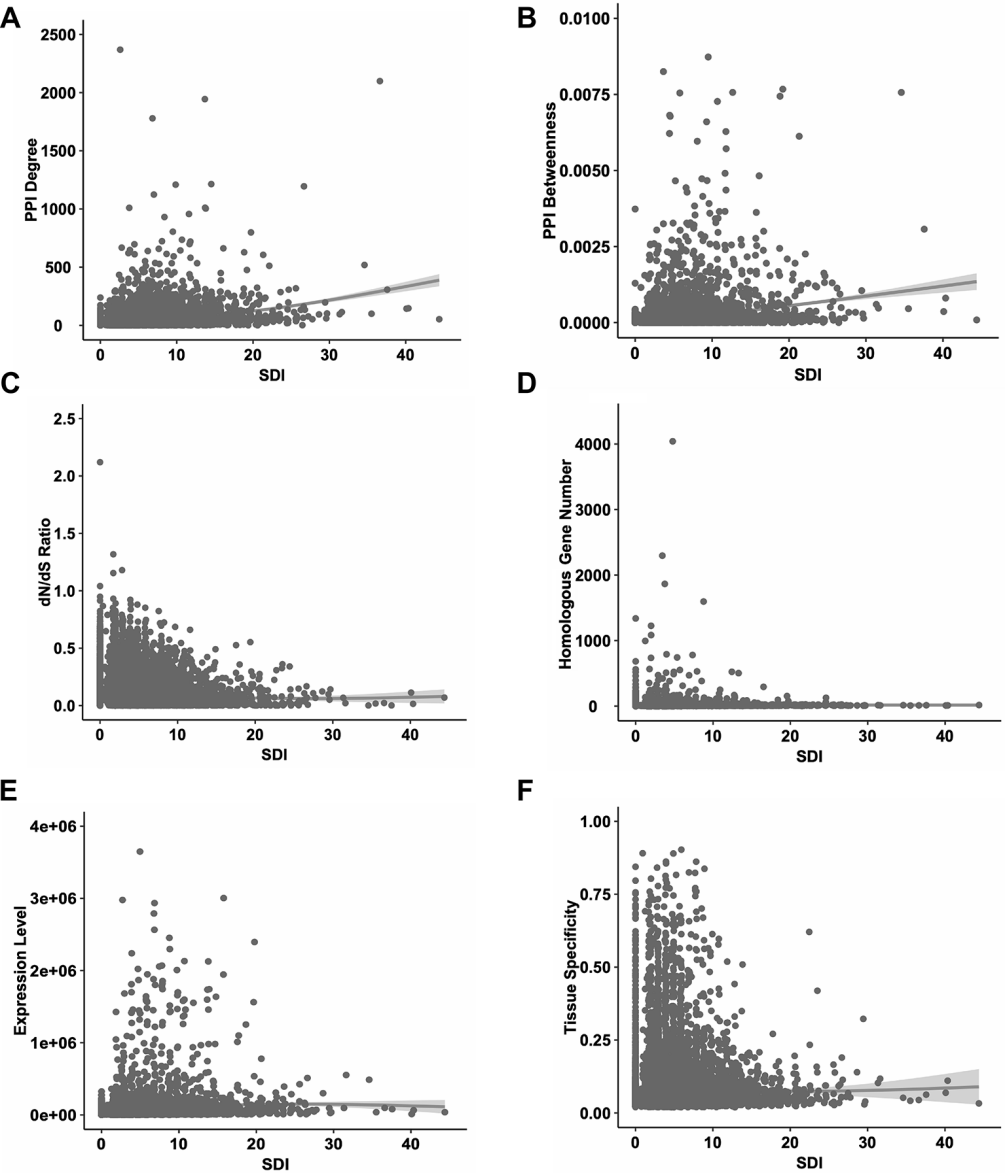
The correlations between SDI and other known measures of essentiality in human genes. The scatter plots show the correlations between SDI and other measures. Genes with higher SDI tend to have higher PPI degrees **(A)**; higher PPI betweenness **(B)**; lower evolutionary rate measured by dN/dS ratio **(C)**; higher homologous gene number **(D)**; higher expression level **(E)** and higher tissue specificity **(F)**. SDI, Subcellular Diversity Index; PPI, protein–protein interaction.

Firstly, we performed correlation analyses for SDI and well-established metrics revealing gene essentiality from three dimensions, PPI topology, conservation and expression patterns (**Figure 2**). As a result, SDI was significantly correlated with those scores [ρ = 0.35, *P* = 0; ρ = 0.36, *P* = 0 with PPI degree and betweenness (14,113 genes) respectively, ρ = -0.20, *P* =7.08e-132 with dN/dS ratio (14,254 genes), ρ = 0.22, *P* =1.02e-181 with homologous gene number (16,696 genes), ρ = 0.21, *P* = 4.91e-156 with expression level, and ρ = 0.12, *P* = 7.29e-52 with tissue specificity (15,167 genes), Spearman's correlation tests]. The correlation results suggest SDI may contribute to gene essentiality as a complement of other measures.

### SDI Performed Well in Predicting Essential Genes

**Figure 3:**
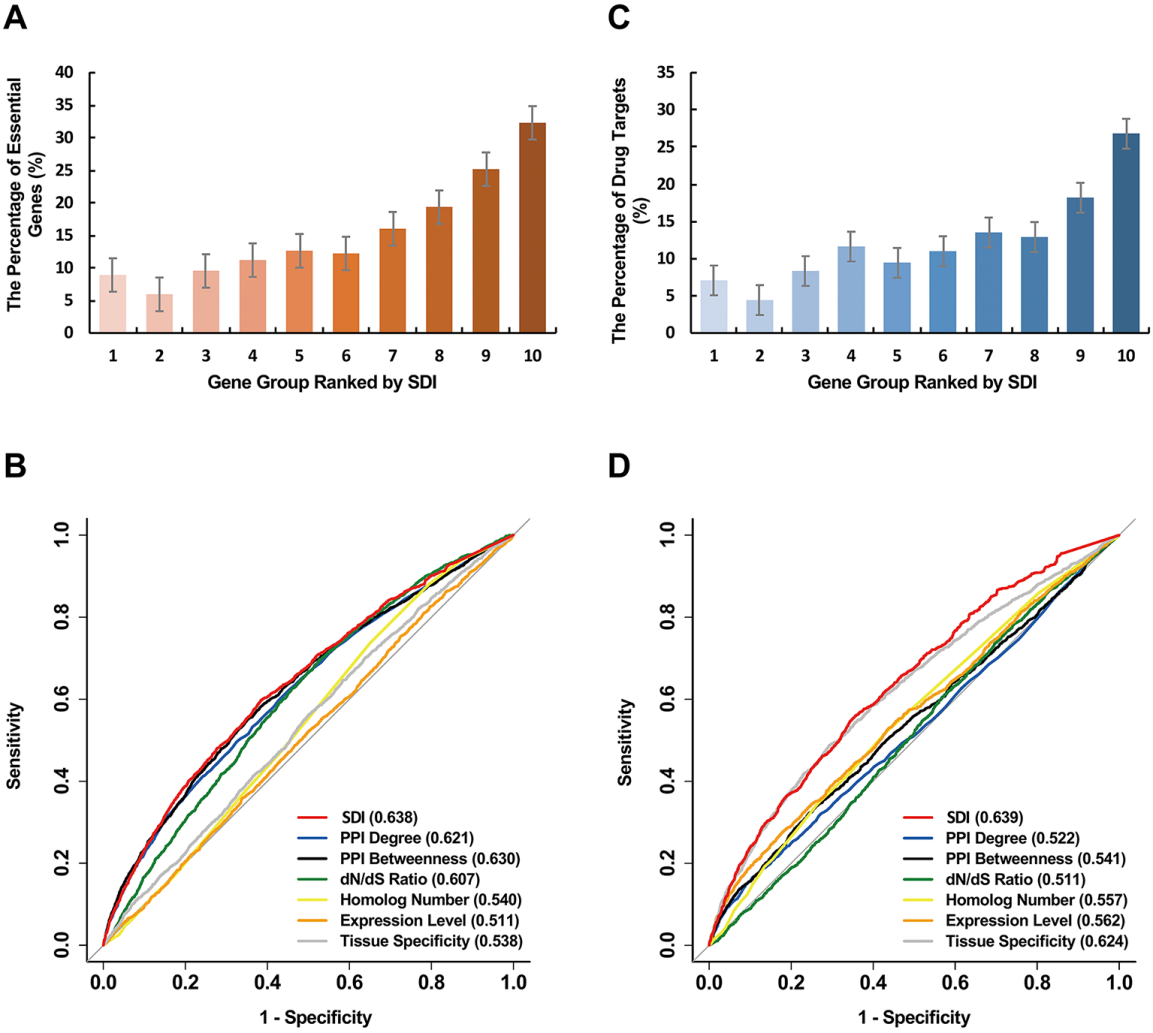
Validation of SDI in human essential genes. The human genes were equally divided into ten groups ranked by SDI. The number of the human essential genes **(A)**, and the human drug targets **(C)** are shown in the bar graphs. The ROC curves show the results from sensitivity tests for validating the human essential genes **(B)** and the human drug targets **(D)**. The sensitivity tests were performed on 11,355 genes. The AUC scores are presented in the brackets. SDI, Subcellular Diversity Index; ROC, receiver operating characteristic; AUC, Area Under Curve.

**Figure 4:**
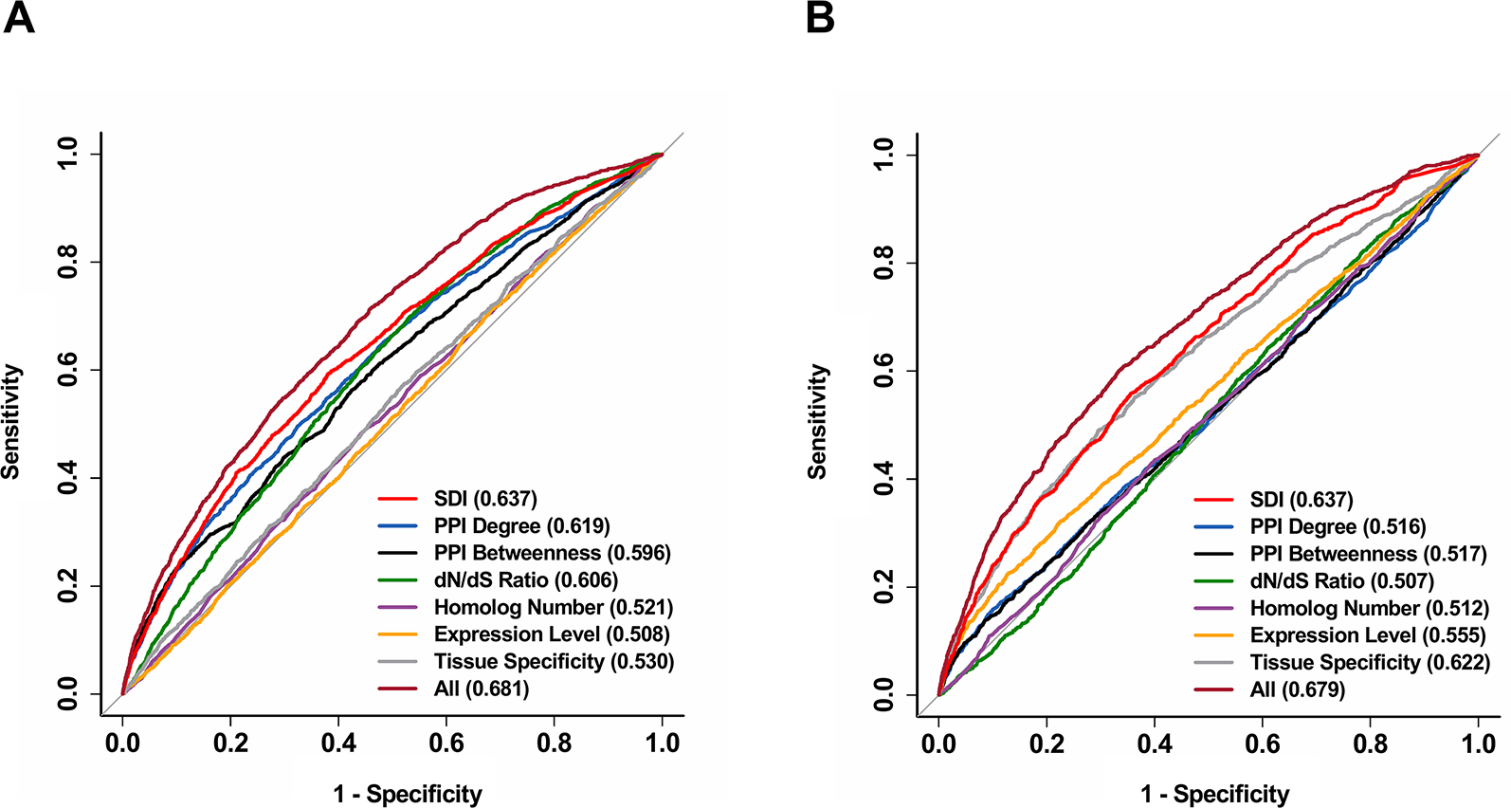
Further validation for the performance of SDI in predicting human essential genes and drug targets. The ROC curves show the results from 10-fold cross-validation of the logistic regression models in predicting human essential genes **(A)** and drug targets **(B)**. All features were used in separate regression models, and a model including all available features was also provided for both essential genes and drug targets. The ROC curves were performed on 11,355 genes. The AUC scores are presented in the brackets. ROC, receiver operating characteristic; AUC, Area Under Curve.

We further explored the correlation of SDI and gene essentiality. Firstly, comparison analyses were performed for the number of essential genes and other genes in ten equally divided human gene groups ranked by SDI. The number of essential genes was observed to increase gradually between the sixth to the tenth group (**Figure 3A**; *P* = 1.19e-140, Pearson's Chi-squared test). Then, we evaluated the prediction ability of SDI using human essential genes from DEG10 (Luo et al., 2014). The result showed that SDI had a good performance in validating the essential genes in 19,134 human genes (AUC = 0.702). To compare the performance of SDI with other metrics, validation tests for SDI and other scores were performed in 11,355 human genes (with all metrics available). As a result, SDI showed the best performance in comparison with other well-established metrics (**Figure 3B**; AUC = 0.638), which suggests its competitive performance in defining gene essentiality. Besides, we performed the prediction and validation of human essential genes by all metrics separately and the integration of all metrics, using logistic regression models and 10-fold cross-validations. SDI was observed to have better performance than other six metrics using the regression models separately (AUC = 0.637), but the model which integrated all metrics had the best performance (AUC = 0.681, **Figure 4A**). This result suggests that SDI and other metrics can reflect gene essentiality from different aspects.

The human essential gene list that we used above contains multiple well-acknowledged essential genes and it may have a certain of bias. Therefore, we further estimate the performance of SDI and other metrics in validating essential genes based on two unbiased datasets from genome-scale essential gene screening experiments in human cell lines (Hart et al., 2015; Tsherniak et al., 2017). SDI shows steady performance in these two datasets (**Figure S3**).

In addition, we also performed the validation tests in the other species to get a better insight. In 6,190 mouse genes with the annotation of SDI, PPI degree and PPI betweenness scores, SDI showed a slightly weaker performance (**Figure S4A**; AUC = 0.627) than PPI degree (AUC = 0.657) and betweenness (AUC = 0.643). For the other 5 species, the results vary from species, yet generally, SDI gives a good performance (**Figure S4B**). The difference in results may be due to different gene number and the number of essential genes mapped to the data for each species.

### SDI May Reveal Druggable Targets of Human Genes

Gene essentiality information has been used to determine the prioritization of novel drug targets or predicting gene druggability (Hu et al., 2007; Radusky et al., 2014). Therefore, we further explored the ability of SDI in revealing gene druggablity. In the top 30 human genes with the highest SDI score, 15 genes are listed as approved drug targets. The 15 unidentified drug targets are mostly phosphoproteins and with products localized in membrane (**Table 2**).

Similar to essential genes, genes with higher SDI scores are more likely to be drug targets (**Figure 3C**; *P* = 7.84e-101, Pearson's Chi-squared test). For validation tests of drug target prediction, SDI performed well in 19,134 human genes (AUC = 0.704). Compared with other measures in overlap genes, SDI showed the best performance (**Figure 3D**, AUC = 0.639).

In addition, we validated the above result by performing the prediction and validation of drug targets by all metrics separately, using logistic regression models and 10-fold cross-validations. SDI was observed to have the best performance among all separate prediction models (AUC = 0.637). Similar to essential genes, the performance became better when all features are used as independent variables in one regression model compared to separate models (AUC = 0.679, **Figure 4B**), indicating the difference between SDI and other metrics in interpretation contributing to the prediction of drug targets. Overall, SDI reveals its ability in predicting drug targets, suggesting its potential in defining gene druggability.

### Drug Targets With Higher SDI Tend to Cause More Side-Effects

**Figure 5:**
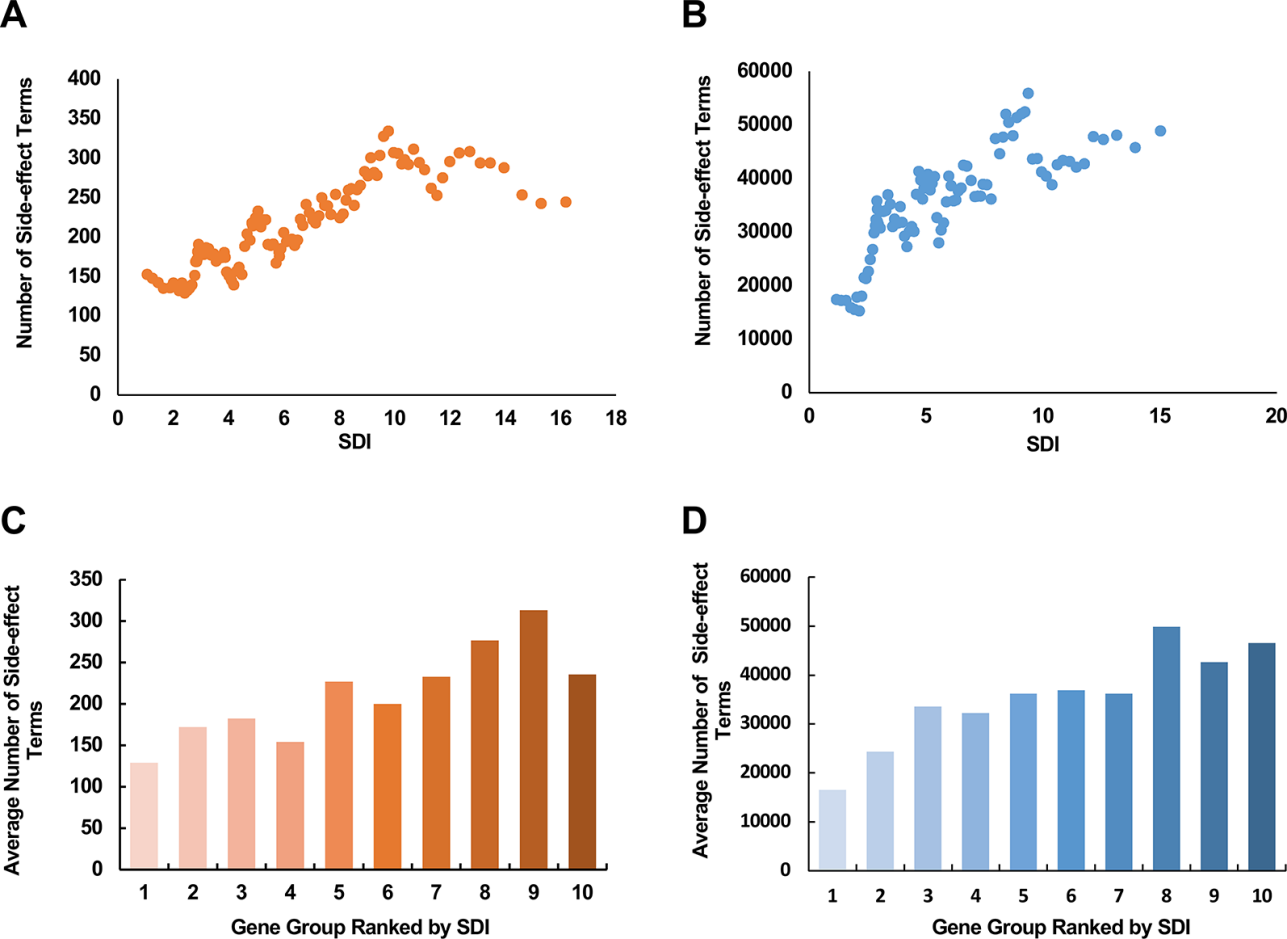
The relationship between SDI and the potential side-effects associated with drug targets. The scatter plots show the correlation between SDI and number of side-effect terms from SIDER database **(A)**; VigiAccess **(B)** of drug targets. The paired scores were smoothed through adequate window and step size. The involved drug target genes were equally divided into ten groups ranked by SDI, and calculated for average numbers of side effect terms from SIDER **(C)** and VigiAccess **(D)**. SDI, Subcellular Diversity Index.

In the pharmaceutical industry, a number of the market withdrawals were caused by drug toxicity (Schuster et al., 2005). Assessing the potential severity of side-effects that a drug target may cause can make great contributions in areas like drug design and clinical drug selection. The subcellular diversity of drug targets seems like one reasonable cause, accounting for the difference in severity of the side-effects that they are responsible for. Under this hypothesis, we explored the relationship between SDI and the number of side-effect terms of a corresponding drug target. We observed a moderate correlation between SDI and term number of side-effects in both data [**Figures 5A**, **B**; ρ = 0.26, *P* = 6.13e-7 with SIDER data (369 genes in total), and ρ = 0.24, *P* = 1.36e-10 with data from VigiAccess (712 genes in total), Spearman's correlation tests]. In addition, average number of side-effects of genes in each of ten equally divided groups ranked by SDI were calculated (**Figures 5C**, **D**). There was a tendency for the average number of side-effect terms to rise between groups in both data. These results suggest SDI may shed new light in estimating poor outcome in drug research.

### Sensitivity Analysis

To examine the robustness of SDI, we randomly chose 5% to 20% of human genes and mouse genes, and changed their GO-CC term into random terms range from 1–45 as the range of actual GO-CC counting of human genes. The performance of SDI in validating essential genes and drug targets only suffered limited loss after manually replacing the actual GO-terms into random GO-terms (**Table 3**). This result indicates the robustness of the SDI algorithm.

**Table 3:** Sensitivity analyses show the robustness of the SDI algorithm.

Human Essential genes
Actual SDI	Test Group	5% Fake Data	10% Fake Data	20% Fake Data
0.702	Group 1	0.685	0.671	0.635
	Group 2	0.687	0.667	0.644
	Group 3	0.682	0.668	0.641
	Average	0.685	0.669	0.64
**Mouse Essential genes**				
**Actual SDI**	**Test Group**	**5% Fake Data**	**10% Fake Data**	**20% Fake Data**
0.695	Group 1	0.686	0.673	0.659
	Group 2	0.680	0.675	0.651
	Group 3	0.687	0.677	0.655
	Average	0.684	0.675	0.655
**Human Drug Targets**				
**Actual SDI**	**Test Group**	**5% Fake Data**	**10% Fake Data**	**20% Fake Data**
0.704	Group 1	0.687	0.675	0.629
	Group 2	0.688	0.674	0.637
	Group 3	0.685	0.673	0.636
	Average	0.687	0.674	0.634

The table shows AUC scores of ROC curves for each test. Sensitivity analyses were performed on 19,341 human genes or 18,499 mouse genes. SDI, Subcellular Diversity Index.

**Figure 6:**
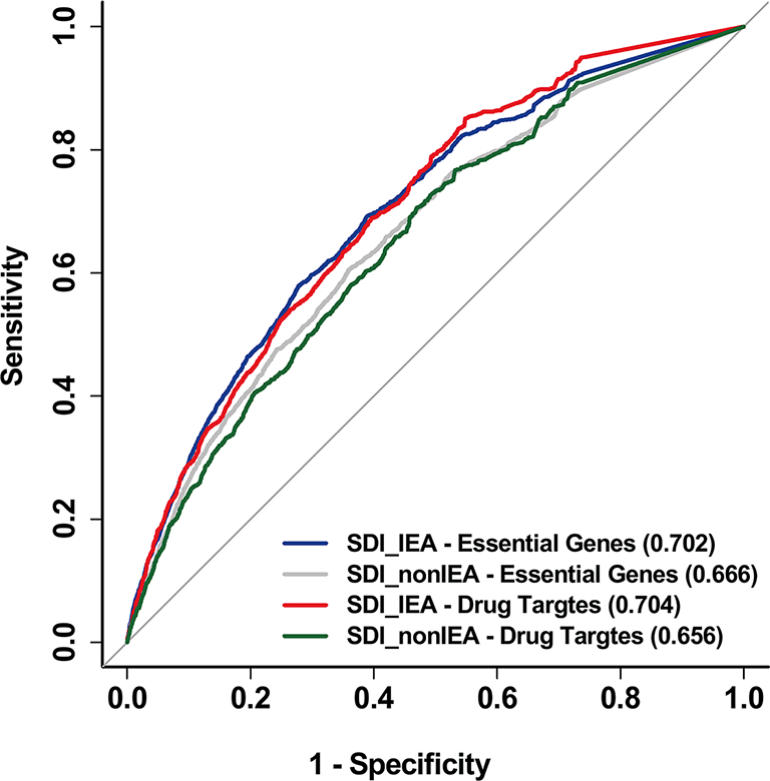
SDI based on non-IEA data was proved to be less powerful than IEA included data. The ROC curves show the performance of SDI based on non-IEA and IEA-included data in validating human essential genes and drug targets. The tests are performed in 19,341 human genes. The AUC scores are presented in the brackets. SDI, Subcellular Diversity Index; ROC, receiver operating characteristic; AUC, Area Under Curve; IEA, Inferred from Electronic Annotation.

In addition, to reduce the bias in gene annotation, we considered all evidence codes included in GO, including the Inferred from Electronic Annotation (IEA) evidence code when performing the SDI algorithm. The SDI calculated by GO-CC data with the removal of IEA annotation proved to be less powerful in performance than IEA included algorithm (**Figure 6**).

## Discussion

Gene annotations in GO-CCO are mainly used for querying for a specific gene, or performing the enrichment analysis of a gene set. The semantic similarity measures of GO terms are also used to reduce redundancy for enrichment analysis in most cases. Our work on SDI was intended to create a measurement of gene characteristic in the context of subcellular locations of gene products, and in the meantime, to make full use of the abundant annotation data and well-developed methods from a different perspective.

Apart from profiling the compartments that gene products localized in as previous studies did, the localization diversity is another way to evaluate the subcellular characteristic of a gene, and it is more convenient for scoring genes at a genomic level. The high performance of SDI in validation of the essential genes and drug targets suggests its applicability in assessing potentially important genes in various aspects.

Based on the annotation data, SDI has some limitations. For example, it can only be performed on genes with annotations, which are mainly protein-coding genes, and it may have a certain kind of bias. However, compared to the other two domains of GO, the GO CC data has less research bias, and the result of unbiased essential gene datasets and sensitivity analyses both confirmed the robustness of SDI. With the increasing interest in the subcellular annotation of genes, the SDI method can be developed to be more robust in the future.

Though limitations exist, our data highlight that the current SDI reveals in part determinants of gene essentiality and druggability. Therefore, the SDI can be used as another gene characteristic in screening and predicting potential genes of importance, to complement other known measurements, which will potentially make contributions to biology and medicine.

## Data Availability Statement

All datasets generated for this study are included in the article/**Supplementary Material**, and the online database of SDI (http://www.cuilab.cn/sdi).

## Author Contributions

QC conceived the project. YZ provided methodological support. KJ and YZ collected the data. KJ performed the computational analysis and wrote the manuscript. QC thoroughly revised the manuscript. All authors discussed the results and contributed to the final manuscript.

## Funding

This work has been supported by grants from the Special Project on Precision Medicine under the National Key R&D Program (2016YFC0903003), the National Natural Science Foundation of China (81670462, 31801099).

## Conflict of Interest

The authors declare that the research was conducted in the absence of any commercial or financial relationships that could be construed as a potential conflict of interest.
