# Supplementary material for: Quantifying Gene Essentiality Based on the Context of Cellular Components
Source: Front Genet. 2020 Jan 21;10:1342. doi: 10.3389/fgene.2019.01342 (PMC6985572; doi:10.3389/fgene.2019.01342)
Supplement: Supplementary file 1 [file DataSheet_1.docx]

Supplementary Material

# Supplementary Figures and Tables

## Supplementary Figures


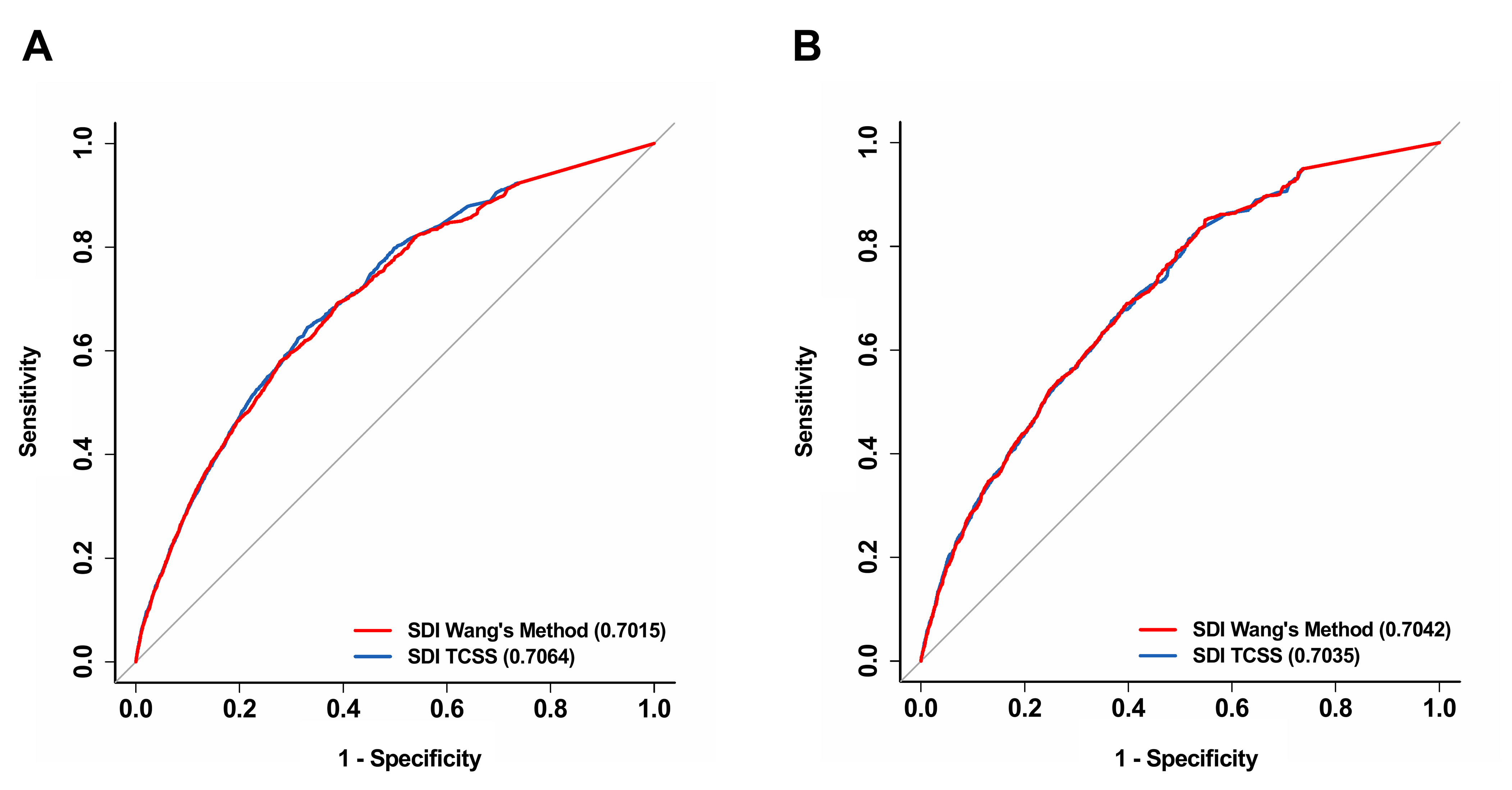


**Supplementary Figure 1.** **The comparation of the performance of SDI using Wang’s method and TCSS method.**

Sensitivity tests were performed on 19,341 human genes in validation of human essential genes (A) and drug targets (B). SDI with TCSS method has better performance in validating essential genes, whereas Wang’s method has better performance in validating drug targets.

**Supplementary Figure 2.** **The comparation of the performance of SDI in validating human essential genes using different average methods.**

Sensitivity tests were performed on 19,341 human genes. Of the three average methods, Root Mean Square (RMS) shows the best performance. M stands for arithmetic mean, and G stands for geometric mean.


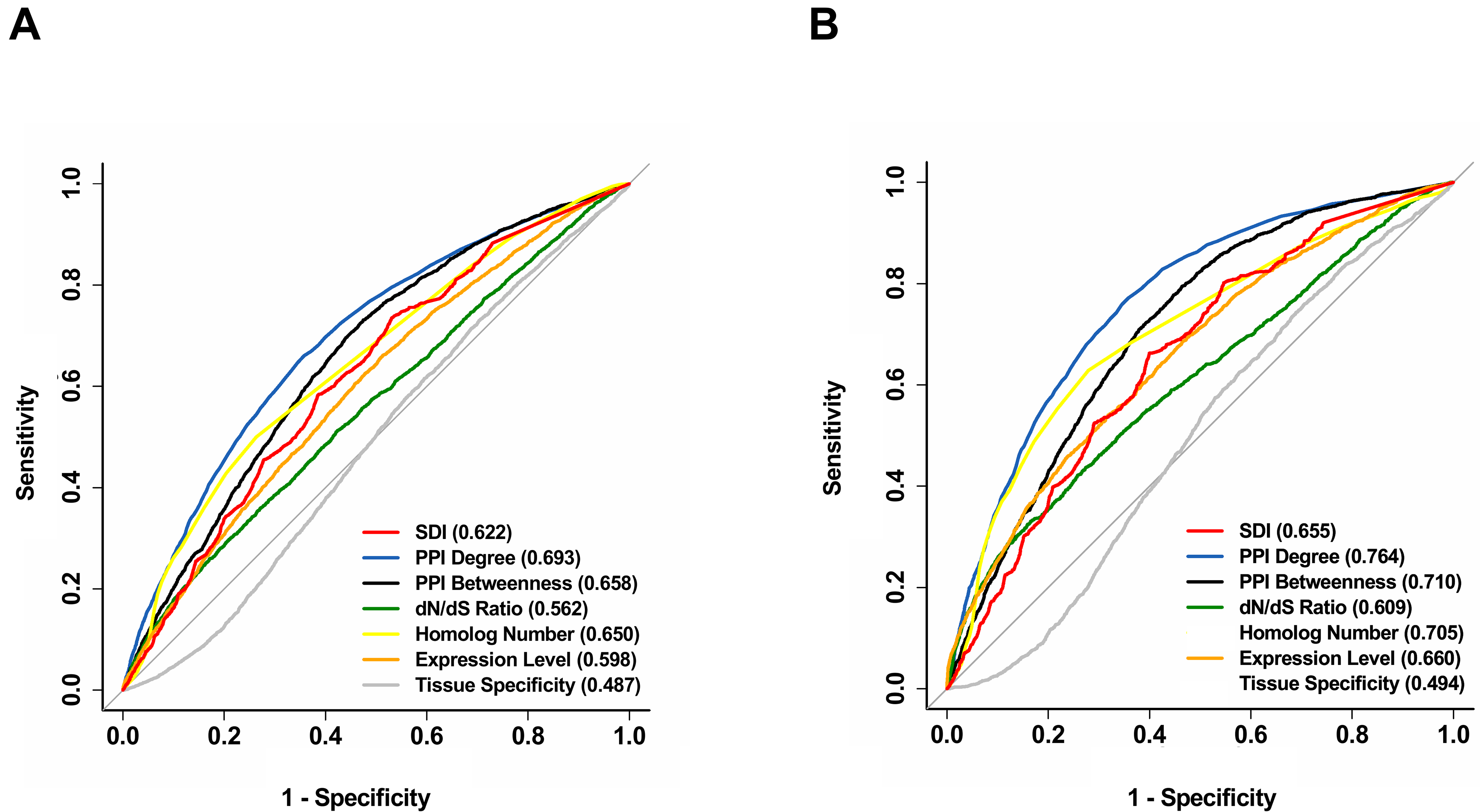


**Supplementary Figure 3.** **Validation of SDI using unbiased essential gene datasets.**

ROC curves show the performance of SDI and all other metrics in validating essential genes from the dataset of Hart *et al.* (A) and DepMap (B).


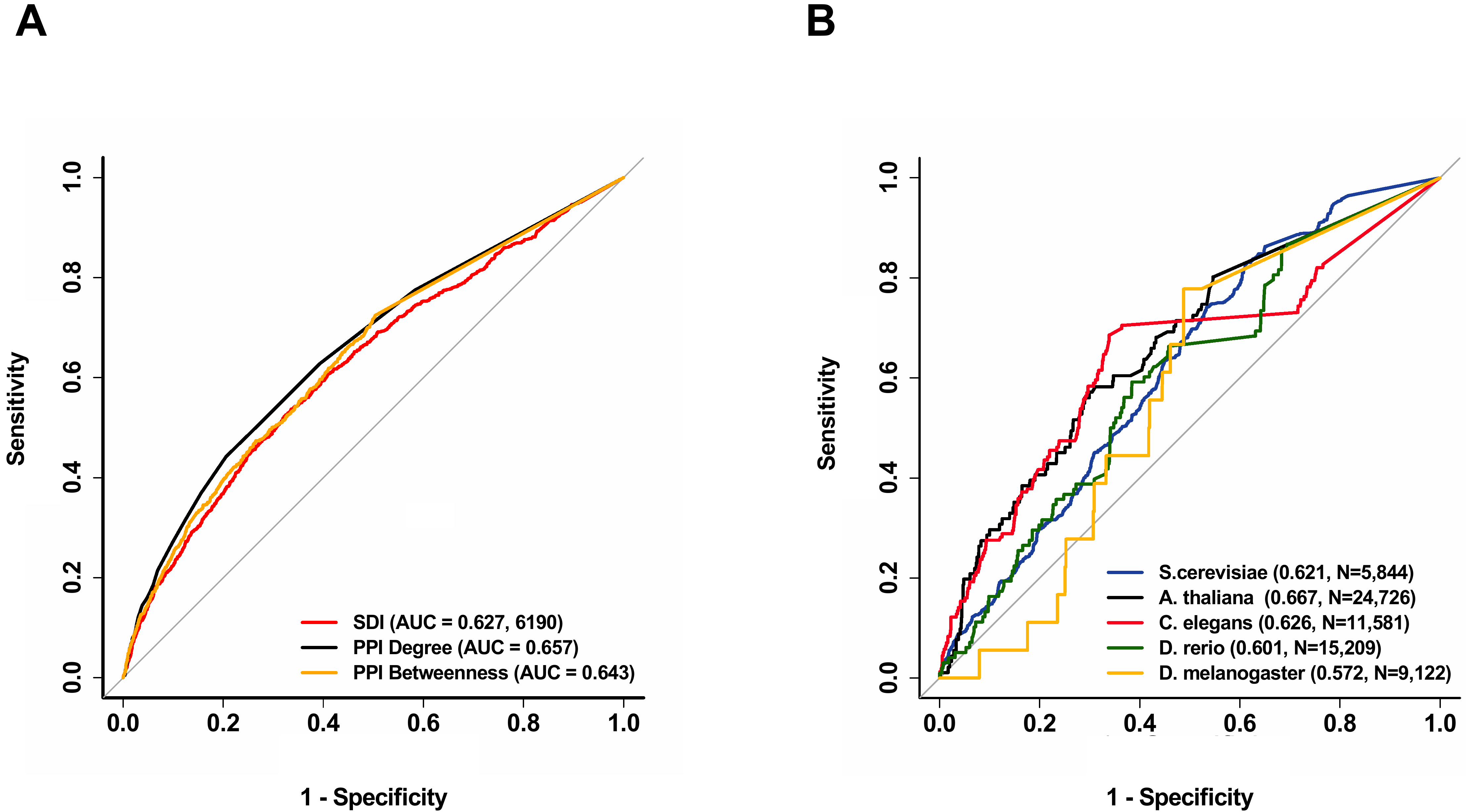


**Supplementary Figure 4.** **Validation of SDI in other species.**

(A) The comparation of the performance of SDI, PPI degree and PPI betweenness in prediction of mouse essential genes (N = 6,190, 1,262 essential genes). (B) The performance of SDI in 5 species in prediction essential genes. The AUC scores and gene number (N) are presented in the brackets (essential gene number: *S.cerevisiae*: 1,084, *A.thaliana*: 182; *C.elegans*: 156; *D.rerio*: 98, *D.melanogaster*: 21).

## Supplementary Tables

**Supplementary Table 1. Contribution factor choice for Wang’s method when implemented the SDI algorithm.**

| **Contribution** | | **Spearman’s Correlation Test** | | | | **AUC** | |  |
| --- | --- | --- | --- | --- | --- | --- | --- | --- |
| **Factor** | |  |  |  |  |  |  |  |
| **Is_a** | **Part_of** | **PPI Degree** | **Expression** | **dN/dS** | **Homologous** | **Essential Genes** | **Drug Targets** | |
|  |  |  | **Level** |  | **Gene** |  |  |  |
|  |  |  |  |  | **Number** |  |  |  |
| 0.8 | 0.6 | 0.354 | 0.214 | -0.203 | 0.22 | 0.702 | 0.704 | |
| 0.7 | 0.5 | 0.357 | 0.214 | -0.203 | 0.22 | 0.702 | 0.703 | |
| 0.9 | 0.7 | 0.351 | 0.213 | -0.203 | 0.219 | 0.701 | 0.705 | |
| 0.6 | 0.8 | 0.359 | 0.214 | -0.204 | 0.22 | 0.702 | 0.702 | |

**Supplementary Table 2. Percentage for the number of GO-CC annotation.**

| **Species** | **1** | **2** | **3** | **4** | **5** | **Gene Number** |
| --- | --- | --- | --- | --- | --- | --- |
| **Mammals** |  |  |  |  |  |  |
| *H. sapiens* | 23.79 | 18.80 | 14.37 | 11.39 | 8.54 | 19341 |
| *M. musculus* | 20.29 | 17.54 | 14.32 | 12.07 | 9.11 | 18499 |
| *R. norvegicus* | 23.82 | 24.72 | 14.36 | 9.99 | 7.05 | 18105 |
|  |  |  |  |  |  |  |
| **Others** |  |  |  |  |  |  |
| *C. elegans* | 23.31 | 46.20 | 13.94 | 6.71 | 3.96 | 11581 |
| *S. cerevisiae* | 15.66 | 19.10 | 16.65 | 14.51 | 12.22 | 5844 |
| *D.melanogaster* | 47.70 | 24.71 | 12.08 | 6.73 | 3.51 | 9122 |
| *D. rerio* | 30.03 | 32.37 | 17.40 | 9.93 | 4.39 | 15209 |
| *A. thaliana* | 44.81 | 24.43 | 13.66 | 7.43 | 3.98 | 24726 |
